# Performance of Early Sepsis Screening Tools for Timely Diagnosis and Antibiotic Stewardship in a Resource-Limited Thai Community Hospital

**DOI:** 10.3390/antibiotics14070708

**Published:** 2025-07-15

**Authors:** Wisanu Wanlumkhao, Duangduan Rattanamongkolgul, Chatchai Ekpanyaskul

**Affiliations:** 1 Department of Adult and Gerontological Nursing, Faculty of Nursing, Srinakharinwirot University, Ongkharak, Nakhon Nayok 26120, Thailand; wisanu.wlk@g.swu.ac.th; 2 Department of Preventive and Social Medicine, Faculty of Medicine, Srinakharinwirot University, Bangkok 10110, Thailand; 3 Division of Multi/Interdisciplinary Studies, Graduate School, Srinakharinwirot University, Bangkok 10110, Thailand

**Keywords:** sepsis screening, early warning scores, antimicrobial resistance, community hospital, low-resource settings, healthcare quality, infectious disease detection

## Abstract

Background: Early identification of sepsis is critical for improving outcomes, particularly in low-resource emergency settings. In Thai community hospitals, where physicians may not always be available, triage is often nurse-led. Selecting accurate and practical sepsis screening tools is essential not only for timely clinical decision-making but also for timely diagnosis and promoting appropriate antibiotic use. Methods: This cross-sectional study analyzed 475 adult patients with suspected sepsis who presented to the emergency department of a Thai community hospital, using retrospective data from January 2021 to December 2022. Six screening tools were evaluated: Systemic Inflammatory Response Syndrome (SIRS), Quick Sequential Organ Failure Assessment (qSOFA), Modified Early Warning Score (MEWS), National Early Warning Score (NEWS), National Early Warning Score version 2 (NEWS2), and Search Out Severity (SOS). Diagnostic accuracy was assessed using International Classification of Diseases, Tenth Revision (ICD-10) codes as the reference standard. Performance metrics included sensitivity, specificity, predictive values, likelihood ratios, and the area under the receiver operating characteristic (AUROC) curve, all reported with 95% confidence intervals. Results: SIRS had the highest sensitivity (84%), while qSOFA demonstrated the highest specificity (91%). NEWS2, NEWS, and MEWS showed moderate and balanced diagnostic accuracy. SOS also demonstrated moderate accuracy. Conclusions: A two-step screening approach—using SIRS for initial triage followed by NEWS2 for confirmation—is recommended. This strategy enhances nurse-led screening and optimizes limited resources in emergency care. Early sepsis detection through accurate screening tools constitutes a feasible public health intervention to support appropriate antibiotic use and mitigate antimicrobial resistance, especially in resource-limited community hospital settings.

## 1. Introduction

Sepsis, a life-threatening condition caused by a dysregulated host response to infection, remains a major global health challenge, particularly in low- and middle-income countries (LMICs) [1,2]. Systematic reviews highlight a disproportionately high burden in LMICs, where limited infrastructure, scarce resources, and delayed diagnoses drive elevated mortality [3]. According to the World Health Organization (2023) [3], sepsis affects about 49 million people annually and causes around 11 million deaths—nearly 20% of global mortality. In Thailand, it is a leading cause of hospital death, with approximately 175,000 cases and 45,000 deaths each year [4]. These figures reflect both the severity of sepsis and the ongoing challenges in timely recognition and management, especially in resource-limited community hospitals.

A global consensus highlights the importance of rapid sepsis identification and intervention, as reflected in the Surviving Sepsis Campaign’s 1 h bundle recommendations [5,6]. However, implementation in rural healthcare systems remains suboptimal due to limited 24 h staffing, inadequate diagnostics, and scarce advanced lab tests [7]. In low-resource settings (LRS), these systemic limitations are even more pronounced. A recent international expert consensus emphasizes the need for context-appropriate adaptations in sepsis care, including reliance on clinical signs (e.g., capillary refill time, mental status, and urine output) when laboratory support is limited, the use of vasopressors via peripheral access when central lines are unavailable, and timely empirical antimicrobial treatment despite limited diagnostics [8]. In Thailand, community hospitals, especially those in rural areas, are key access points where emergency department (ED) nurses often lead initial triage. This nurse-led approach places significant responsibility on frontline nursing staff to recognize early signs of sepsis, making the accuracy and usability of screening tools critically important for timely clinical intervention [9].

Early Warning Scores (EWSs), which assess clinical parameters such as heart rate, respiratory rate, blood pressure, and mental status, have emerged as critical decision-support tools designed to facilitate the prompt identification of deteriorating patients at risk of developing severe outcomes such as sepsis [10]. Various scoring systems have been developed, including the National Early Warning Score (NEWS) [11], the National Early Warning Score 2 (NEWS2) [10], which improves upon NEWS, the Quick Sequential Organ Failure Assessment (qSOFA) score, the Systemic Inflammatory Response Syndrome (SIRS) criteria, the Modified Early Warning Score (MEWS), and the Search Out Severity (SOS) score [5,7,12,13,14,15]. These scoring systems differ significantly in complexity, parameters required, and predictive performance [16,17,18]. The comparison of each screening tool is shown in Table 1.

The SIRS criteria are known for their high sensitivity, effectively identifying early stages of sepsis, although their utility is limited by low specificity, resulting in numerous false positives [19]. In contrast, qSOFA offers greater prognostic specificity and is recognized for its predictive accuracy in identifying patients at high risk of adverse outcomes; however, it is less sensitive, potentially missing early cases that require immediate medical attention [20,21]. NEWS2 incorporates additional refinements, such as a modified oxygen saturation scale tailored for patients with chronic respiratory conditions, and assesses new-onset confusion, improving its capacity for broader clinical risk stratification [10,22]. Despite promising performance data from high-resource settings, NEWS2 remains insufficiently evaluated in LMIC contexts, especially community hospitals with constrained resources [23,24].

Most comparative studies assessing the diagnostic accuracy of sepsis screening tools have been conducted predominantly in urban tertiary hospitals [12,13] and university-affiliated hospitals [9,24,25,26,27], where infrastructure, staffing, and clinical pathways are substantially more robust compared to rural community hospitals [28,29]. Consequently, findings from such urban-based studies may not be fully generalizable to resource-constrained rural contexts prevalent in LMICs. Additionally, the frequent reliance on positive blood cultures as the gold standard for sepsis diagnosis presents significant operational limitations in resource-constrained settings, primarily due to prolonged turnaround times and limited availability of laboratory facilities [30]. Thus, the reliance on blood cultures can lead to considerable delays and hinder timely clinical decision-making, making them impractical for frontline emergency care.

Timely and accurate identification of sepsis is crucial not only for patient survival but also for promoting appropriate antibiotic utilization. Prompt recognition of sepsis helps clinicians initiate targeted antibiotic therapy early, potentially reducing unnecessary broad-spectrum antibiotic use, which is a major contributor to antimicrobial resistance (AMR). Effective sepsis screening, therefore, constitutes a critical public health strategy aimed at optimizing antibiotic stewardship practices in resource-limited settings [3,31,32].

While the Sepsis-2 definition emphasized the presence of systemic inflammation (SIRS) in response to infection [12], the more widely accepted Sepsis-3 criteria redefined sepsis as life-threatening organ dysfunction caused by a dysregulated host response to infection, operationalized as an increase in SOFA score by ≥2 points [19,33,34]. However, both definitions rely on clinical and laboratory assessments that may not be feasible in rural, low-resource settings. Therefore, early warning scores provide a simplified and pragmatic alternative for frontline triage, particularly when used in conjunction with clinical judgment or ICD-coded diagnoses.

Given these limitations, using physician-determined final diagnoses based on the International Classification of Diseases, Tenth Revision (ICD-10) offers a pragmatic, clinically relevant, and immediately actionable alternative reference standard. While not without inherent limitations, ICD-10-coded diagnoses effectively represent the clinical judgments made within the real-world constraints of community hospital practice, thus providing ecological validity essential for evaluating the practical performance of early warning scores [35,36].

The primary objective of this study was to critically evaluate and directly compare the diagnostic accuracy of six early warning scores (SIRS, qSOFA, MEWS, NEWS, NEWS2, and SOS) in identifying sepsis-related diagnoses as determined by ICD-10 coding among patients presenting to the emergency department of a Thai community hospital. The secondary objective was to guide the development of practical nurse-led triage protocols designed explicitly for rural, resource-limited emergency care environments, using ICD-10 diagnosis codes as a pragmatic reference standard. Moreover, establishing accurate screening protocols contributes directly to enhancing antibiotic stewardship and reducing antimicrobial resistance by minimizing unnecessary antibiotic exposure through precise early detection and targeted therapy [5,18,31].

## 2. Results

### 2.1. Patient Selection and Demographic Characteristics

**Figure 1:**
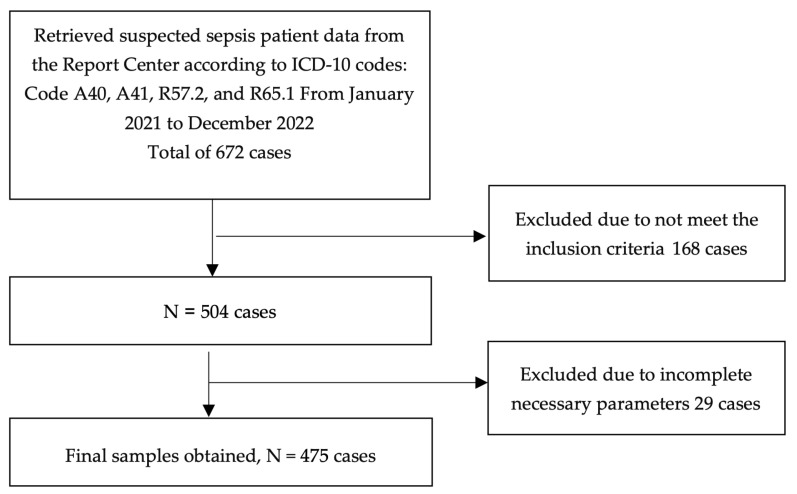
Flowchart of patient selection for final analysis.

Initially, 672 patients suspected of having sepsis were identified from hospital records between January 2021 and December 2022. Of these, 168 patients were excluded for not meeting the inclusion criteria—specifically, patients under 18 years of age, those referred to other hospitals without admission, and repeat visits by the same individual within 28 days after discharge. An additional 29 patients were excluded due to incomplete medical records. Thus, a total of 475 patients were included in the final analysis (Figure 1).

The study population is shown in Table 2. Most were male patients (51.79%), aged 61–75 years (36.63%), with a mean age of 65.35 years (SD = 17.01). About 80% of the patients had underlying diseases, with hypertension (49.89%), dyslipidemia (38.32%), and diabetes mellitus (25.68%) being the most common. Other frequently reported conditions included cardiovascular disease (13.05%) and chronic kidney disease (10.53%). The most frequent final ICD-10-based diagnosis was sepsis (50.95%), followed by pneumonia (16.21%), urinary tract infection (UTI) (9.26%), and cellulitis (5.68%). The overall mortality rate at the end of treatment in this hospital was 4.0%.

Of the 475 patients, one-fifth had positive blood culture results (25.89%). Most primary sources in these patients were Gram-negative bacilli strains, such as *Escherichia coli*. Details of demographic and clinical characteristics, as well as blood culture results, are shown in Table 2.

### 2.2. Diagnostic Accuracy of Early Warning Scores

The diagnostic accuracy of six early warning scores (SIRS, qSOFA, MEWS, NEWS2, NEWS, and SOS) was evaluated using ICD-10 diagnoses as the reference standard for sepsis.

SIRS criteria showed the highest sensitivity at 84% (95% CI: 80–89%) but had a low specificity of only 25% (95% CI: 20–31%). Its positive predictive value (PPV) and negative predictive value (NPV) were 54% and 61%, respectively. The AUROC was 0.578 (95% CI: 0.527–0.630), indicating a high risk of false-positive results.

qSOFA yielded the highest specificity at 91% (95% CI: 88–95%) and the highest PPV at 66% (95% CI: 53–78%). However, it had a very low sensitivity of 16% (95% CI: 11–20%) and an AUROC of 0.577 (95% CI: 0.525–0.628), reflecting its limited utility as a primary screening tool due to a high false-negative rate.

MEWS showed a more balanced performance, with a sensitivity of 69% (95% CI: 63–75%) and specificity of 48% (95% CI: 42–55%). The AUROC was 0.623 (95% CI: 0.573–0.673), and its PPV and NPV were 58% and 60%, respectively.

NEWS2 achieved moderate diagnostic accuracy with 68% sensitivity and 52% specificity. Its AUROC of 0.625 (95% CI: 0.575–0.675) indicated slightly improved discriminative capacity over other tools.

NEWS yielded a sensitivity of 67% (95% CI: 61–73%) and specificity of 53% (95% CI: 46–59%), with an AUROC of 0.624 (95% CI: 0.574–0.674). Its predictive values were comparable to those of NEWS2.

SOS score demonstrated moderate specificity at 67% (95% CI: 61–73%) but lower sensitivity at 51% (95% CI: 45–58%). The AUROC was 0.619 (95% CI: 0.569–0.670), with PPV and NPV of 62% and 57%, respectively, as shown in Table 3.

**Figure 2:**
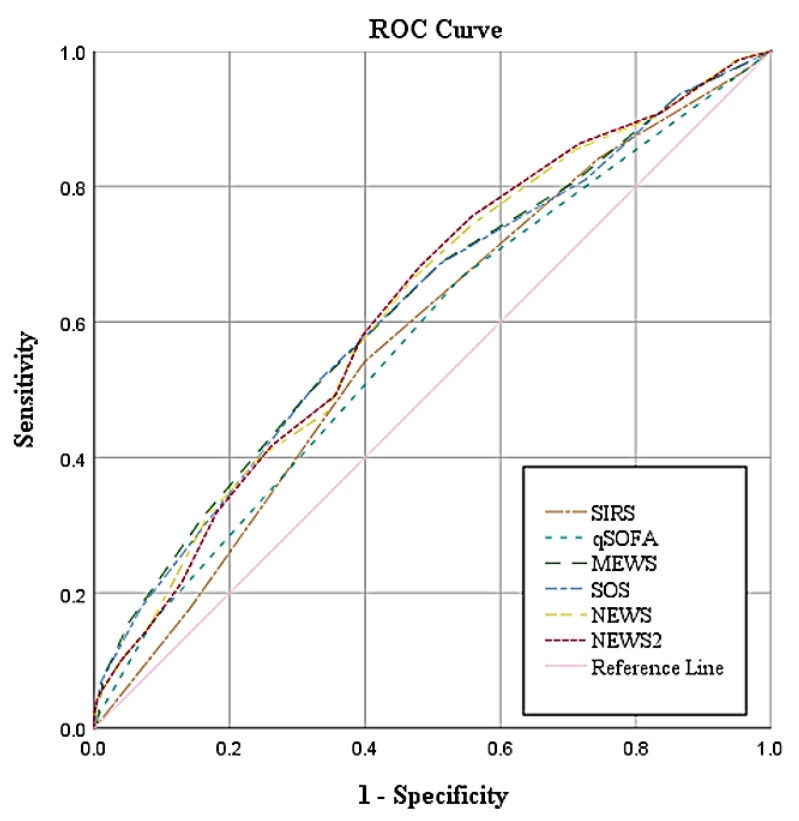
Comparison of ROC curves for six sepsis screening tools using ICD-10 as the reference standard.

The ROC curves visually confirmed these findings, illustrating a clear trade-off between sensitivity and specificity across different scores. NEWS2 showed the highest overall discriminative ability, closely followed by NEWS and MEWS, suggesting these tools might offer optimal frontline screening potential within resource-limited settings (Figure 2).

### 2.3. Summary of Findings

Overall, the analysis indicated no single early warning score provides ideal diagnostic accuracy for sepsis identification in isolation. SIRS criteria effectively identified most true-positive sepsis cases but at the cost of many false positives. Conversely, qSOFA, with its high specificity, could effectively confirm sepsis but missed numerous cases. NEWS2, NEWS, and MEWS demonstrated more balanced accuracy, suggesting their practical application in nurse-led initial triage and risk assessment in community hospitals with constrained resources.

These results support the potential utility of a two-step screening approach, involving the initial use of a high-sensitivity tool (such as SIRS) for broad triage, followed by a high-specificity tool (such as qSOFA) for diagnostic confirmation, to optimize resource utilization and clinical effectiveness.

## 3. Discussion

The main finding of this study is that a two-step screening approach using SIRS followed by NEWS2 provides a balanced and practical method for early sepsis identification in a resource-constrained Thai community hospital. This study critically assessed the diagnostic accuracy of six early warning scores (SIRS, qSOFA, MEWS, NEWS2, NEWS, and SOS) using ICD-10-coded diagnoses as a pragmatic reference standard for identifying sepsis in a Thai community hospital setting characterized by resource constraints. The findings confirm previous studies suggesting that no single early warning score alone achieves optimal diagnostic accuracy, highlighting the need for a strategic combination of screening tools in practice [23,24].

Consistent with previous research, the current study found that SIRS demonstrated the highest sensitivity (84%), reinforcing its utility in initial broad screening for early signs of sepsis, aligning closely with several earlier studies conducted in both high-resource and low-resource environments [16,17,19]. However, its notably low specificity (25%) significantly increased false-positive rates, which poses substantial challenges in resource-limited hospitals by potentially overwhelming healthcare resources with unnecessary diagnostic and therapeutic interventions [37].

In line with previous international and regional studies, qSOFA showed superior specificity (91%) but was associated with low sensitivity (16%) [19,38]. This mirrors findings by Singer et al. (2016) [19] and further validation studies by Tusgul et al. (2017) [38], indicating qSOFA’s role primarily as a prognostic tool rather than as an initial screening measure due to its risk of missing early-stage sepsis cases.

The current findings showed that NEWS2 offered balanced performance (AUROC = 0.625), while being moderately sensitive (68%) and specific (52%). These outcomes are consistent with findings from other studies that have evaluated NEWS2 across different contexts, including high-income and middle-income countries, reflecting its broad clinical applicability and balanced accuracy for initial sepsis identification [22,39]. Conversely, MEWS and NEWS demonstrated similar moderate diagnostic accuracies (AUROC = 0.623 and 0.624, respectively), aligning with research from Thailand and internationally that recommended these tools as reliable options for frontline sepsis screening due to their simplicity and ease of use by nursing staff [13,40].

The SOS score, specifically developed for Thai clinical settings, displayed moderate accuracy (AUROC = 0.619), consistent with prior local studies. However, the relatively lower sensitivity (51%) compared to SIRS or NEWS2 underscores the limitation of SOS in capturing initial subtle clinical deterioration, highlighting a need for cautious interpretation when applied as a standalone tool [14,36].

Importantly, effective early detection of sepsis through such screening methods contributes directly to antibiotic stewardship. Early recognition enables targeted antibiotic therapy, reducing unnecessary broad-spectrum antibiotic use and mitigating antimicrobial resistance, a critical public health challenge in hospital settings [5,18,31].

Although our study was conducted in the emergency department of a small community hospital, the importance of structured screening and response mechanisms in resource-limited settings is broadly supported in the literature. For example, Kovacevic et al. (2021) [41] reported that implementing a structured checklist (CERTAIN) in a low-resource ICU improved care processes, such as reducing the duration of antibiotics and mechanical ventilation, even though mortality outcomes remained unchanged. This suggests that systematic approaches to sepsis management, whether in ER or ICU settings, can enhance treatment efficiency in LRS contexts [41]

### 3.1. Rationale for Two-Step Screening Approach: SIRS Followed by NEWS2

Given the varying strengths and limitations of these tools, this study strongly advocates a two-step approach rather than relying on a single tool. Contrary to the traditional recommendation using SIRS followed by qSOFA, our data and local clinical context support a strategy employing SIRS as an initial screening tool due to its high sensitivity, followed by NEWS2 as a confirmatory measure.

Firstly, NEWS2, unlike qSOFA, encompasses multiple physiological parameters (respiratory rate, oxygen saturation, supplemental oxygen use, systolic blood pressure, heart rate, consciousness level, and temperature), providing more comprehensive clinical information crucial for effective patient management decisions by frontline nurses, who often operate independently in community hospitals [10,39,42]. This advantage is critical in contexts where medical resources and physician availability are limited, necessitating robust clinical tools that support independent nurse-led triage and decision-making [32,39].

Secondly, NEWS2 has demonstrated consistent performance in identifying early clinical deterioration in various patient populations, including sepsis, pneumonia, and COVID-19, indicating its versatility and effectiveness beyond sepsis identification alone [43,44,45]. In contrast, the restricted parameter scope of qSOFA—comprising mental status, respiratory rate, and systolic blood pressure—limits its ability to detect subtle and early signs of deterioration, as highlighted in recent validation studies [19,31].

Additionally, the continuous monitoring potential offered by NEWS2 allows clinicians and nurses to promptly reassess patient conditions and respond dynamically, particularly critical in community hospitals where patient conditions can rapidly evolve [22,46]. This continuous reassessment capability is notably absent in qSOFA, reinforcing NEWS2’s practical superiority in real-world clinical practice. Implementing this two-step approach can thus represent an innovative public health intervention by systematically promoting judicious antibiotic utilization, directly addressing antimicrobial resistance at the point of care.

### 3.2. Strengths, Limitations, and Practical Considerations

This study’s strengths lie in its real-world setting, substantial sample size (*n* = 475), and use of ICD-10 coding, which reflects routine clinical diagnoses in resource-limited environments. These factors enhance ecological validity and relevance to similar global contexts. However, several limitations should be noted. The retrospective design may lead to biases in data completeness and accuracy. ICD-10-coded diagnoses, though pragmatic, may lack the precision of biological markers [35]. Furthermore, diagnostic coding practices may vary between clinicians or institutions, potentially leading to inconsistencies in sepsis classification and data interpretation. The single-center nature of the study also limits generalizability, underscoring the need for multi-center validation. Delays in hemoculture results due to limited resources [47] and subjective assessments of parameters like consciousness and respiratory rate [48] may further affect reliability.

Future studies should address these limitations and incorporate advanced technologies, such as rapid diagnostics and artificial intelligence (AI), to improve sepsis screening and antibiotic stewardship. AI-enhanced NEWS2 could offer more accurate and practical screening tools [49]. Prospective evaluation of the two-step protocol’s impact on antibiotic use and antimicrobial resistance is warranted. In clinical practice, especially in community hospitals, nurses are central to early sepsis detection and response. Effective implementation of NEWS2 requires appropriate training, clear protocols, and continuous monitoring to ensure improved patient outcomes and safety [10,11].

## 4. Materials and Methods

### 4.1. Study Design and Setting

This cross-sectional study was conducted at Maewong Hospital, a 30-bed community hospital located in Nakhon Sawan Province, Thailand. The hospital serves a predominantly rural population and provides essential emergency care under resource-limited conditions.

The hospital’s ED is nurse-led and operates 24 h a day with support from physicians primarily during daytime hours. The ED handles approximately 70–100 patients daily, including trauma, medical, and surgical emergencies. Staffing consists of 12 registered nurses, 1 emergency medical technician (EMT), and 3 nurse aides, working in rotating 8 h shifts (morning, evening, night). In cases of critical illness, such as severe sepsis or septic shock, patients are stabilized and transferred to a higher-level provincial hospital. The ED operates with limited access to advanced laboratory and imaging facilities and relies heavily on clinical criteria and early warning scores for initial triage and management decisions. This setting reflects the typical constraints faced by many rural Thai hospitals, making it a suitable context for evaluating the practical performance of sepsis screening tools.

### 4.2. Population and Sampling

The study included adult patients (≥18 years) who presented to the emergency department at Maewong Hospital with suspected sepsis between January 2021 and December 2022. Patients were identified through hospital records using ICD-10 codes corresponding to sepsis (A40, A41), severe sepsis (R57.2), and septic shock (R65.1), as documented in the clinical diagnoses. The sample size was calculated based on the area under the receiver operating characteristic (AUROC) curve values from similar sepsis studies, using an estimated AUROC of 0.7, an alpha level of 0.05, and 80% power. The required minimum was approximately 450 cases; the final sample size of 475 ensured adequate statistical power for diagnostic accuracy analysis.

### 4.3. Data Collection and Instruments

Data were collected using a structured case record form in two parts: Part 1 included demographic and clinical information of the patients, such as gender, age, comorbidities, final diagnoses based on ICD-10 codes, and hemoculture results. Part 2 was a recording form for the parameters of the various screening tools under comparison: NEWS2, NEWS, qSOFA, SIRS, MEWS, and SOS scores. The parameters of each tool are shown in Table 1, except for MEWS and SOS, which did not include urine output in their calculations due to the unavailability of this information in the emergency department but still utilized the same cut points as if the urine output parameter were included. For multiple entries in one parameter, the first recorded data in the emergency room were selected for the case record form. The accuracy of each parameter and the case record form was verified by three experts: an epidemiologist, an infectious disease physician, and an experienced nurse from the emergency department.

The primary reference standard for identifying sepsis was the physician-confirmed final diagnosis using ICD-10 codes, which reflect the clinical diagnosis documented in patient records. The relevant ICD-10 codes included A40, A41, R57.2, and R65.1, representing sepsis and septic shock-related conditions. This pragmatic reference was selected due to common limitations in community hospitals, such as delays in laboratory confirmation, including blood culture results.

### 4.4. Statistical Analysis

All data were entered and analyzed using SPSS for Windows (version 26.0; IBM Corp., Armonk, NY, USA). Descriptive statistics, including frequencies and percentages, were used to summarize the data. To evaluate the predictive accuracy of the sepsis screening tools, diagnostic scores were calculated, using ICD-10-coded diagnoses as the reference standard.

Diagnostic performance was assessed through several key measures. Sensitivity was calculated as the number of patients with positive scores divided by the number of patients diagnosed with sepsis according to ICD-10 codes, multiplied by 100. Specificity was determined by dividing the number of patients with negative scores by the number of patients not diagnosed with sepsis by ICD-10 codes, then multiplying by 100. The positive predictive value (PPV) represented the proportion of patients diagnosed with sepsis among those with positive screening scores, while the negative predictive value (NPV) reflected the proportion of patients not diagnosed with sepsis among those with negative scores. Both values are expressed as percentages and presented together with 95% confidence intervals.

To further assess test performance, the positive likelihood ratio (LR+) was calculated by dividing sensitivity by one minus specificity, and the negative likelihood ratio (LR−) was computed by dividing one minus sensitivity by specificity. Finally, the AUROC curve was used to evaluate the overall discriminatory ability of each screening tool. The ROC curve was plotted with sensitivity (true-positive rate) on the *Y*-axis and one minus specificity (false-positive rate) on the *X*-axis.

## 5. Conclusions

This study evaluated the diagnostic accuracy of six early warning scores—SIRS, qSOFA, MEWS, NEWS, NEWS2, and SOS—using ICD-10-coded diagnoses as a practical reference standard in a Thai community hospital. SIRS showed the highest sensitivity, while qSOFA offered the highest specificity. NEWS2, along with NEWS, MEWS, and SOS, demonstrated limited but relatively better accuracy for initial triage in resource-constrained settings. Based on these findings, a recommended two-step screening approach using SIRS followed by NEWS2 not only supports timely, nurse-led decision-making and resource optimization, but also represents a strategic public health intervention to improve antibiotic stewardship and reduce antimicrobial resistance. Implementation of such strategies could significantly enhance patient outcomes and public health in low-resource hospital settings. Given its simplicity and feasibility, the approach may also be adapted for use in other low- and middle-income countries (LMICs) with similar emergency care constraints, thereby expanding its relevance and potential global impact.

## Figures and Tables

**Table 1:** Comparison of the various sepsis screening tools and their key attributes.

Criteria	NEWS	NEWS2	qSOFA	SIRS	MEWS	SOS
**Parameters**	RR, O2 saturation and supplement SBP, HR, Level of consciousness, (AVPU) Temperature,	RR, O2 saturation and supplement SBP, HR, Level of consciousness, (ACVPU) Temperature	RR, SBP, Altered mental status	RR, HR, Temperature, WBC count	RR, SBP, HR, Level of consciousness (AVPU), Temperature,	RR, SBP, HR, Level of consciousness (ACVPU), Temperature, Urine output
**Score Range**	0–20	0–20	0–3	≥2 out of 4	0–14	0–16
**Thresholds**	≥5	≥5	≥2 out of 3	≥2 out of 4	≥4	≥4 Varies by institution
**Pros**	Incorporates oxygen use, validated for diverse settings	Widely used, straightforward, SpO_2_ Scale 2 for COPD	Easy to calculate, identify sepsis risk for pre-hospital setting	Long-standing use, well understood	Simple, widely used	Specific to sepsis-related organ failure
**Cons**	More complex than qSOFA or SIRS	More complex than qSOFA or SIRS	Less sensitive than SIRS	Can be non-specific, many false positives	May not be specific to sepsis	More complex than qSOFA and SIRS
**Advantages over Others**	Includes oxygen saturation adjustments	Established benchmarks, broad applicability	Rapid, quick assessment	Captures early signs of systemic response	Early identification of deterioration	Focused on sepsis-related organ dysfunction

**Table 2:** Demographic, clinical characteristics and blood culture results of patients.

Characteristic	Number of Patients	Percentage (%)
Gender		
Male	246	51.79
Female	229	48.21
End of Treatment		
Alive	456	96.00
Dead	19	4.00
Age (years)		
18–30	17	3.58
31–45	56	11.79
46–60	86	18.10
61–75	174	36.63
>75	142	29.90
Comorbidity Diseases		
No	95	20.00
Yes	380	80.00
1 comorbidity	99	20.84
2 comorbidities	106	22.32
3 comorbidities	94	19.79
4 comorbidities	58	12.21
5 comorbidities	18	3.79
6 comorbidities	5	1.05
Chronic Diseases		
Hypertension	237	49.89
Dyslipidemia	182	38.32
Diabetes mellitus	122	25.68
Heart disease	62	13.05
Chronic kidney disease	50	10.53
Gout	45	9.47
Chronic obstructive pulmonary disease/	43	9.05
Asthma		
Benign prostatic hyperplasia	39	8.21
Cerebrovascular disease	33	6.95
Liver disease	26	5.47
Cancer	26	5.47
Tuberculosis	20	4.21
Human Immunodeficiency Virus	14	2.95
Epilepsy	10	2.11
Anemia	8	1.68
Others	31	6.53
Diagnosis		
Sepsis	242	50.95
Pneumonia	77	16.21
UTI	44	9.26
Cellulitis	27	5.68
Fever unspecified	19	4.00
Infection diarrhea	17	3.58
Bronchitis	8	1.69
Cutaneous abscess	8	1.69
Dengue	7	1.47
Bacterial infection	6	1.26
Others	20	4.21
Blood Culture Results		
Positive	123	25.89
Negative or no growth	352	74.11
Type of Bacteria from Hemoculture (n = 102) *		
*Escherichia coli*	47	46.08
*Staphylococcus aureus*	10	9.80
*Klebsiella pneumoniae*	8	7.85
*Streptococcus* group A	8	7.85
*Streptococcus viridans* group	7	6.86
*Burkhoderia pseudomallei*	3	2.94
*Aeromonas caviae*	2	1.96
*Kocuria marina*	2	1.96
*Staphylococcus saprophyticus*	2	1.96
*Streptococcus pneumoniae*	2	1.96
*Streptococcus* group D	2	1.96
*Enterobacter cloacae*	1	0.98
*Morganella morganii (CRE)*	1	0.98
*Paenibacillus* spp.	1	0.98
*Proteus mirabilis*	1	0.98
*Pseudomonas aeruginosa*	1	0.98
*Salmonella* spp.	1	0.98
*Streptococcus mutans*	1	0.98
*Streptococcus suis*	1	0.98
*Streptococus* group B	1	0.98

* After excluding *Staphylococcus epidermidis* 16 cases, *Acinetobacter lwoffii* 1 case, *Bacillus* spp. 1 case, *Bacillus subtilis* 1 case, *Coronybacterium* spp. 1 case, yeast cells 1 case.

**Table 3:** Diagnostic accuracy of six sepsis screening tools (SIRS, qSOFA, MEWS, NEWS2, NEWS, and SOS) compared to ICD-10 as the reference standard.

Sepsis Screening Tools	%Sensitivity (95%CI)	%Specificity (95%CI)	%Positive Predictive Value (95%CI)	%Negative Predictive Value (95%CI)	Positive Likelihood Ratio	Negative Likelihood Ratio	Area Under ROC Curve (95%CI)
SIRS	84 (80–89)	25 (20–31)	54 (49–59)	61 (51–71)	1.12	0.64	0.578 (0.527–0.630)
qSOFA	16 (11–20)	91 (88–95)	66 (53–78)	51 (46–56)	1.78	0.92	0.577 (0.525–0.628)
MEWS	69 (63–75)	48 (42–55)	58 (52–64)	60 (53–67)	1.33	0.65	0.623 (0.573–0.673)
NEWS2	68 (62–74)	52 (62–74)	60 (54–65)	61 (54–68)	1.42	0.62	0.625 (0.575–0.675)
NEWS	67 (61–73)	53 (46–59)	59 (54–65)	60 (54–67)	1.43	0.62	0.624 (0.574–0.674)
SOS	51 (45–58)	67 (61–73)	62 (55–68)	57 (51–63)	1.55	0.73	0.619 (0.569–0.670)

## Data Availability

The original contributions presented in this study are included in the article. Further inquiries can be directed to the corresponding authors.

## Abbreviations

The following abbreviations are used in this manuscript:

ACVPU	alert, confusion, voice, pain, unresponsive
AMR	antimicrobial resistance
AUROC	area under receiver operating characteristic
AVPU	alert, voice, pain, unresponsive
COPD	chronic obstructive pulmonary disease
CI	confidence interval
ED	emergency department
EMT	emergency medical technician
HR	heart rate
ICD-10	International Classification of Diseases, Tenth Revision
LMICs	low- and middle-income countries
LR+	positive likelihood ratio
LR−	negative likelihood ratio
LRS	low-resource settings
MEWS	Modified Early Warning Score
NEWS	National Early Warning Score
NEWS2	National Early Warning Score version 2
NPV	negative predictive value
O2	oxygen
PPV	positive predictive value
qSOFA	Quick Sequential Organ Failure Assessment
RR	respiratory rate
ROC	receiver operating characteristic
SBP	systolic blood pressure
SIRS	Systemic Inflammatory Response Syndrome
SpO_2_	peripheral oxygen saturation
SOS	Search Out Severity
UTI	urinary tract infection
